# Examination of the characteristics of long-term survivors among patients with gallbladder cancer with liver metastasis who underwent surgical treatment: a retrospective multicenter study (ACRoS1406)

**DOI:** 10.1186/s12876-022-02234-9

**Published:** 2022-03-28

**Authors:** Ryota Higuchi, Hiroaki Ono, Ryusei Matsuyama, Yusuke Takemura, Shinjiro Kobayashi, Takehito Otsubo, Yuta Abe, Itaru Endo, Minoru Tanabe, Masakazu Yamamoto, Association for Clinical Research on Surgery Group (ACRoS1406)

**Affiliations:** 1 Department of Surgery, Institute of Gastroenterology, Tokyo Women’s Medical University, 8-1, Kawada-cho, Shinjuku-ku, Tokyo, 162-8666 Japan; 2 Department of Hepatobiliary and Pancreatic Surgery, Tokyo Medical and Dental University, 1-5-45 Yushima, Bunkyo-ku, Tokyo, 113-8519 Japan; 3 Department of Gastroenterological Surgery, Yokohama City University, Graduate School of Medicine, 3-9 Fukuura, Kanazawa-ku, Yokohama, 236-0004 Japan; 4 Department of Surgery, Keio University School of Medicine, 35 Shinanomachi, Shinjuku-ku, Tokyo, 160-8582 Japan; 5 Division of Gastroenterological and General Surgery, St. Marianna University School of Medicine, 2-16-1 Sugaro, Miyamae-ku, Kawasaki, Kanagawa 216-8511 Japan

**Keywords:** Neoplasms, Metastasis, Surgery

## Abstract

**Background:**

Gallbladder cancer (GBC) with liver metastasis is considered unresectable. However, there have been infrequent reports of long-term survival in patients with GBC and liver metastases. Therefore, we examined the characteristics of long-term survivors of gallbladder cancer with liver metastasis.

**Methods:**

A retrospective multicenter study of 462 patients with GBC (mean age, 71 years; female, 51%) was performed. Although patients with pre-operatively diagnosed GBC and liver metastasis were generally excluded from resection, some cases identified during surgery were resected.

**Result:**

In patients with resected stage III/IV GBC (n = 193), the period 2007–2013 (vs. 2000–2006, hazard ratio 0.63), pre-operative jaundice (hazard ratio 1.70), ≥ 2 liver metastases (vs. no liver metastasis, hazard ratio 2.11), and metastasis to the peritoneum (vs. no peritoneal metastasis, hazard ratio 2.08) were independent prognostic factors for overall survival, whereas one liver metastasis (vs. no liver metastasis) was not. When examining the 5-year overall survival and median survival times by liver metastasis in patients without peritoneal metastasis or pre-operative jaundice, those with one liver metastasis (63.5%, not reached) were comparable to those without liver metastasis (40.4%, 33.0 months), and was better than those with ≥ 2 liver metastases although there was no statistical difference (16.7%, 9.0 months). According to the univariate analysis of resected patients with GBC and liver metastases (n = 26), minor hepatectomy, less blood loss, less surgery time, papillary adenocarcinoma, and T2 were significantly associated with longer survival. Morbidity of Clavien–Dindo classification ≤ 2 and received adjuvant chemotherapy were marginally not significant. Long-term survivors (n = 5) had a high frequency of T2 tumors (4/5), had small liver metastases near the gallbladder during or after surgery, underwent minor hepatectomy without postoperative complications, and received postoperative adjuvant chemotherapy.

**Conclusions:**

Although there is no surgical indication for GBC with liver metastasis diagnosed pre-operatively, minor hepatectomy and postoperative chemotherapy may be an option for selected patients with T2 GBC and liver metastasis identified during or after surgery who do not have other poor prognostic factors.

**Supplementary Information:**

The online version contains supplementary material available at 10.1186/s12876-022-02234-9.

## Background

Gallbladder cancer (GBC) is the most common biliary malignancy and the sixth most common gastrointestinal malignancy, with a prevalence rate ranging from 8.5 cases per 100,000 population in the United States (US) to 13.6 cases per 100,000 in Asian populations [1]. GBC occurs relatively often in certain geographic locations, such as Chile, North India, South Korea, Japan, and New Mexico (US) [2]. GBC is known to have a poor prognosis as it is often found during its early metastatic stage [3]. It is associated with fewer chemotherapy regimens than other cancers, and surgery is the only curative treatment that can be expected to confer long-term survival in patients with GBC [4]. In particular, the median survival time of patients with stage IVB GBC and distant metastasis is very poor, at 2–7 months [3, 5, 6]. In clinical practice guidelines, stage IVB GBC is not indicated for resection [4].

However, in line with this concept, oligometastasis (defined as the intermediate state of spread, now synonymous with isolated distant metastases) in long-term survivors of other carcinomas with distant metastases has been reported [7–10]. There have also been infrequent reports of long-term survival in patients with GBC and distant metastases, including liver metastases [11–13]. However, there are no reports of oligometastasis in patients with GBC. The outcome of surgical resection in patients with GBC oligometastasis, especially liver metastasis, is not well understood. In patients in whom small liver metastases are found during radical resection and in the absence of other distant metastases, on pre-operative images, whether surgery should be performed is uncertain.

## Methods

We investigated the characteristics of patients and long-term survivors of GBC and oligomestases, especially liver metastases. Patients aged 20 years or older with pathologically diagnosed GBC were included. We did not consider the stage of the disease, previous treatment, or tumor resection. The exclusion criterion was the patient’s refusal to participate in the research through opportunities provided for refusal during information disclosure. We also excluded patients with insufficient clinical and/or histopathologic data and pathological types other than adenocarcinoma.

We identified 462 patients with pathological GBC diagnosed between 2000 and 2013 at five university hospitals: Tokyo Medical and Dental University (Tokyo, Japan [n = 89]), Yokohama City University (Kanagawa, Japan [n = 87]), Keio University (Tokyo, Japan, [n = 68]), St. Marianna University (Kanagawa, Japan [n = 25]), and Tokyo Women’s Medical University (Tokyo, Japan [n = 193]). We included all the patients with GBC diagnosed during the target period. The median age of this patient sample was 71 years (range 35–91), and 237 (51%) patients were female.

### Surgical approach

Multidisciplinary clinical approaches, including pre-operative and post-operative indications, varied by institution. However, the basic surgical strategy was as follows. Patients with distant metastases based on pre-operative diagnosis were generally excluded from resection. Furthermore, when pre-operative examination showed suspected GBC, and complete resection with negative margin (R0) resection was considered possible, surgical resection was indicated. The number of liver metastases found intra-operatively was based on macroscopic findings and intra-operative ultrasonography. Successful R0 resection was defined as hepatectomy, bile duct resection, lymph node dissection, and additional resection of the surrounding organs in order to secure a surgical margin. Decisions regarding hepatectomy, the extent of lymph node dissection, and resection of surrounding organs were made based on the policy of each institution. In some cases, small liver and/or distant lymph node metastases revealed during surgery were resected at the discretion of the surgeon at each institution, based on the extent of the surgical resection, the degree of invasiveness of the surgery, and the safety of surgical treatment. For incidental GBC, additional resections, such as additional liver resection and/or bile duct resection or pancreaticoduodenectomy and/or lymph node dissection, were considered, either intra-operatively or in two stages based on the extent of GBC. Gallbladder bed resection and resection of segments 4a and 5 of the liver were defined as minor hepatectomy, and hepatectomy of three or more segments, such as right hepatectomy, was defined as major hepatectomy.

### Pathological examination

All specimens were assessed for tumor progression by pathologists at each institution (rather than centralized). Formalin-fixed paraffin-embedded tissue sections were examined histologically according to the 8th edition of the American Joint Committee on Cancer (AJCC 8) staging system [14], and the 6th edition of the General Rules for Surgical and Pathological Studies on Cancer of the Biliary Tract of the Japanese Society of Biliary Surgery [15]. This included observations regarding primary tumor status, lymph node involvement, and histopathological grade (Additional file 1: Table S1). Liver metastases were defined as follows: H0, no liver metastases; H1, one liver metastasis; and H2, two or more liver metastases. The metastatic site was pathologically confirmed.

Small liver metastasis was defined as liver metastasis measuring less than 5 mm in size. This was done since most metastases that cannot be identified using current diagnostic imaging are 5 mm or less in size.

### Neoadjuvant and adjuvant chemotherapy and follow-up

Neoadjuvant and adjuvant chemotherapy were administered based on facility standards (Additional file 1: Table S2). Postoperative recurrence was evaluated using regular computed tomography (CT) scan and ultrasonography (performed once every 3 months for 2 years, and once every 6 months for years 2–5) and by the detection of tumor markers (once every 1–3 months for 2 years, and once every 6 months for years 2–5 years). Appropriate chemotherapy was considered if tumor recurrence was confirmed**.** The time of recurrence and death were assessed. Loss to follow-up was defined as a case in which the patient’s vital status (alive/dead) could not be confirmed for more than 2 years, except for patients who lived for more than 5 years, or who were known to have died by the time of study data collection in March 2019.

### Statistical analysis

Between-group differences in the qualitative and quantitative variables were determined using two-tailed Fisher’s exact and Wilcoxon rank sum tests, respectively. Survival analyses were performed using the Kaplan–Meier method, log-rank tests, and Cox proportional hazards models. Only factors that were significant in the univariate analysis were included in the multivariate analysis. In the multivariate analysis, we used reference values for carbohydrate antigen (CA) 19.9 and carcinoembryonic antigen (CEA) levels, and for the surgery time and blood loss, the median was used as the cut-off value. Due to the advancement in medical care year on year, the year of surgery was divided into two periods and analyzed as one of the factors. All statistical analyses were performed using R, version 3.6.1 (R Foundation for Statistical Computing, Vienna, Austria). A p-value ≤ 0.05 was considered statistically significant.

## Results

Resection was performed in 328 patients (71%), and 16% of resection cases were incidental GBC, which we defined as diagnosis during surgery or pathological examination. The surgical mortality rate in patients was 1.5%. In terms of hepatic margins, gallbladder bed resection was performed in 98 (30%), resection of segment 4a plus 5 of the liver (S4aS5 resection) in 50 (15%), resection of three or more liver segments in 62 (21%), and cholecystectomy in 110 (34%) patients. In terms of bile duct margins or lymph node dissection, bile duct resection was performed in 125 (38%) and PD in 31 (10%) patients. The numbers and proportions of Tis or 1/T2/T3/T4 in the T stage were 72 (23%), 112 (34%), 102 (31%), and 41 (13%), respectively. Those of N0/N1/N2/distant lymph node metastasis in the N stage were 200 (61%), 76 (23%), 14 (4%), and 37 (11%), respectively, and with distant metastasis (M1) in M stage was 54 (17%). The overall R0 resection rate was 81%. Details of the clinical and pathological findings are summarized in Additional file 1: Table S2, and details of non-resected cases are summarized in Additional file 1: Table S3.

### *Overall survival at each stage (Fig. **1**)*

**Fig. 1:**
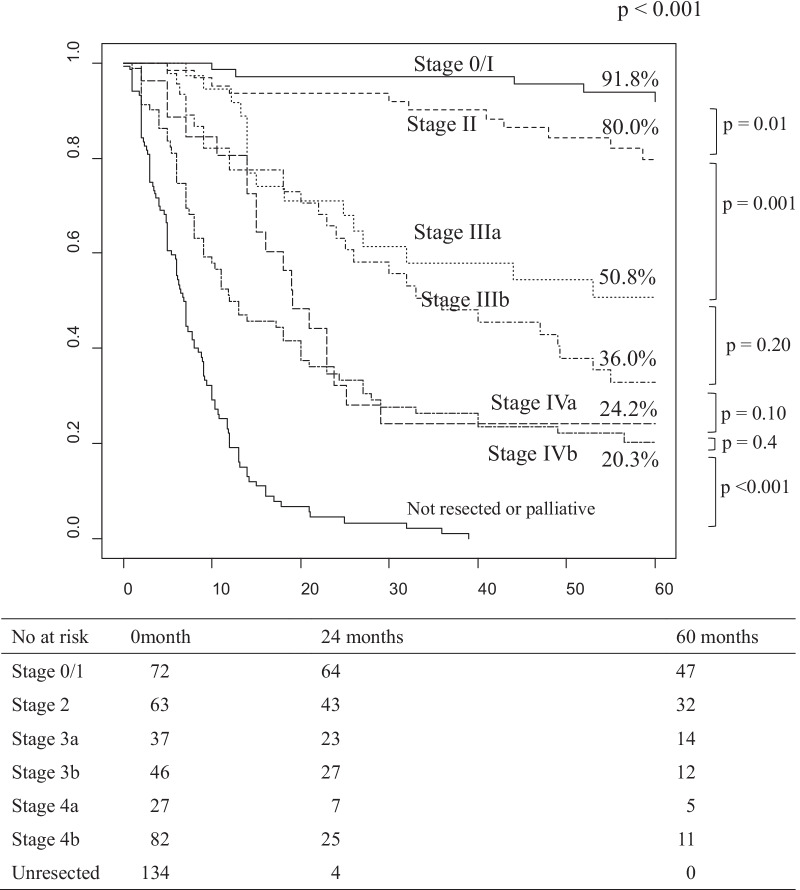
Overall survival rate according to the AJCC 8th stage in patients with gallbladder cancer. Median survival time for stage 0/1, 2, 3a, 3b, 4a, 4b and unresected were "not reached", "not reached", "not reached", 36.0, 19.1, 12.0 and 6.8 months, respectively. No at risk, number at risk

There were 239 deaths from cancer (52%), 22 deaths from other diseases (5.0%), 145 survivors (31%), and 56 lost to follow-up (12%). The median follow-up period was 42 months for the resected cases and 6 months for the non-resected cases. Five-year survival was 91.8%, 80.0%, 50.8%, 36.0%, 24.2%, 20.3% and 0% in patients with stage I/II/IIIA/IIIB/IVA/IVB/unresected GBC, respectively. Comparing the adjacent stages, the differences in survival between patients with stages 0/I and II GBC, between patients with stages II and IIIA GBC, and between patients with stage IVB and non-resected GBC were statistically significant, but there were no significant differences in survival in comparisons between patients in the other adjacent stages. The 5-year survival rate for each stage of each facility and the recurrence rate for each facility were in Additional file 1: Table S2. There was a significant difference between the survival rate of stage IIIb, IVb, and the recurrence rate by institution (Additional file 1: Table S4).

### Prognostic factors for overall survival in patients with stage III or IV GBC

The surgery period 2007–2013 (vs. 2000–2006, hazard ratio [HR] 0.63), pre-operative jaundice (HR 1.70), two or more liver metastasis (vs. no liver metastasis, HR 2.11), metastasis to the peritoneum (vs. no peritoneal metastasis, HR 2.08) were independent prognostic factors for overall survival. The 5-year survival rate was 0% for patients with peritoneal dissemination, compared to 30.1% for those with one liver metastasis (Table 1). One liver metastasis (vs. no liver metastasis) was not an independent prognostic factor for overall survival.

**Table 1:** Uni- and multivariate analyses of overall survival in patients with resected stage III-IV GBC

		Univariate			Multivariate	
		n	OS 5 years	p value^a^	Hazard ratio (95% CI)	p value^b^
Period	07–13′/00–06′	95/98	37.8/21.3	**< 0.001**	0.63 (0.41–0.95)	**0.026**
Age (year)	≥ 70/< 70	96/97	33.3/25.7	0.60		
Sex	Female/male	108/85	27.4/32.1	0.90		
Preop. Jaundice	Yes/no	63/128	12.1/38.0	**< 0.001**	1.70 (1.02–2.86)	**0.044**
NAC	Yes/no	11/182	11.4/30.6	0.30		
CA19-9, U/L	> 37/≤ 37	110/79	16.2/46.6	**< 0.001**	1.25 (0.99–1.32)	0.31
CEA, ng/mL	> 5/≤ 5	59/132	15.2/35.6	**0.003**	1.52 (0.81–1.94)	0.054
Incidental	Yes/no	19/174	9.59/31.3	0.08		
Hepatectomy	Ch/GB	34/47	34.9/39.5	**< 0.001**	1.90 (0.95–3.82)	0.071
	S4aS5/GB	42/47	39.2/39.5		1.21 (0.68–2.15)	0.51
	≥ 3 seg/GB	62/47	11.9/39.5		1.71 (0.93–3.13)	0.082
BDR.PD	Absent/BDR	58/107	38.7/24.1	0.06		
	PD/ BDR	28/107	30.4/24.1			
Blood loss, mL	≥ 864/< 864	91/90	13.5/42.0	**< 0.001**	1.33 (0.79–2.23)	0.29
Surgery time, min	≥ 366/< 366	91/90	18.2/37.9	**0.001**	1.08 (0.66–1.79)	0.76
Histology	pap/tub1.2	23/129	50.2/27.0	**0.02**	0.59 (0.29–1.20)	0.15
	tub3/tub1.2	40/129	23.7/27.0		1.42 (0.84–2.38)	0.19
AJCC T 8th	T2/T3	50/102	43.6/27.4	**< 0.001**	1.30 (0.74–2.27)	0.36
	T4/T3	41/102	16.5/27.4		1.12 (0.65–1.95)	0.67
AJCC N 8th	N0/N1	65/77	39.2/26.1	0.07		
	N2/N1	14/77	9.23/26.1			
	Distant/N1	37/77	25.0/26.1			
H	H1/H0	12/167	30.1/46.3	**0.03**	1.03 (0.43–2.49)	0.94
	H2/H0	14/167	7.69/46.3		2.11 (1.06–4.21)	**0.035**
P	P1.2/P0	16/177	0/32.2	**< 0.001**	2.08 (1.05–4.12)	**0.036**
Residual cancer	R1.2/R0	60/131	12.1/36.9	**< 0.001**	1.29 (0.82–2.02)	0.27
Morbidity*	≥ 3/≤ 2	54/137	18.6/33.8	**0.004**	1.44 (0.94–2.22)	0.097
AC	with /without	86/98	32.8/28.5	0.30		

Bold values indicate *p* < 0.05

OS, overall survival; NAC, Neoadjuvant chemotherapy; CA, cancer antigen; CEA, carcinoembryonic antigen; CI, confidence interval; GB, gallbladder bed resection; Ch, cholecystectomy; S4aS5, resection of segment 4a plus 5 of the liver; BDR, bile duct resection; PD, pancreatoduodenectomy; AJCC, American Joint Committee on Cancer

*Clavien–Dindo classification; AC, adjuvant chemotherapy. pap/tub1/tub2/tub3/other, T2/T3/T4, N0/N1/N2/Distant LNM, H0/H1/H2, P0/P1/P2, R0 (see Additional file 1: Table S1)

^a^Log-rank test

^b^Cox proportional hazards model

### Overall survival and median survival due to liver metastasis in stage III/IV gallbladder cancer patients without peritoneal dissemination nor jaundice (Fig. 2)

**Fig. 2:**
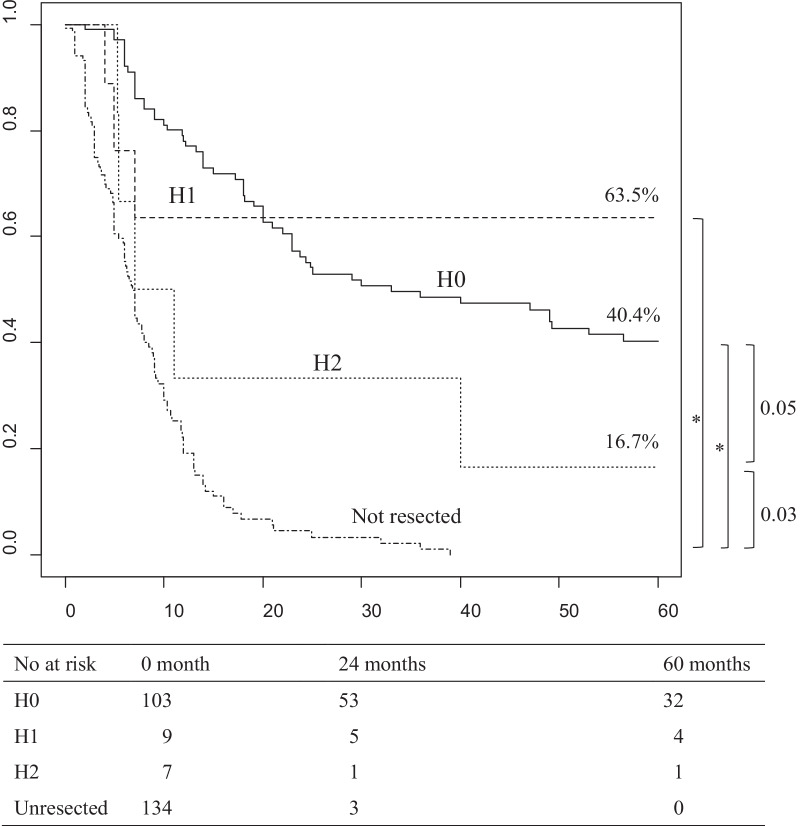
Overall survival due to liver metastasis in stage *III/IV* gallbladder cancer patients without peritoneal dissemination or jaundice. Median survival times for H0, H1, H2, and unresected were 33, not reached, 9.0, and 6.8 months, respectively. *< 0.001, H0, no liver metastasis; H1, one liver metastasis; H2, two or more liver metastasis, No at risk, number at risk

When examined in patients without pre-operative jaundice and peritoneal dissemination, which were significant in multivariate analysis, 5-year overall survival and median survival time were significantly better in H1 patients than in unresected patients but equivalent to H0 patients.

These were significantly worse in H2 patients than in H0 patients.

### *Cumulative recurrence rate due to liver metastasis in stage III/IV gallbladder cancer patients without peritoneal dissemination or jaundice (Fig. **3**)*

**Fig. 3:**
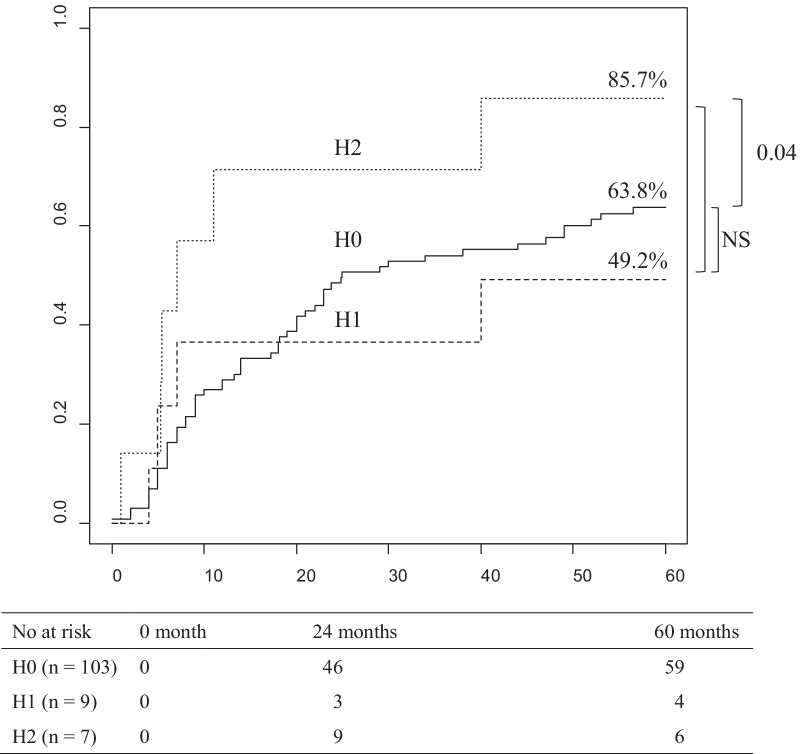
Cumulative recurrence rate due to liver metastasis in stage *III/IV* gallbladder cancer patients without peritoneal dissemination or jaundice. Median time to recurrence for H0, H1, and H2 were 25.0, "not reached," and 7 months, respectively. H0, no liver metastasis; H1, one liver metastasis; H2, two or more liver metastasis; No at risk, number at risk

The cumulative 5-year recurrence rate of H1 patients was equivalent to that of H0 patients and better than that of H2 patients, although the comparison between H1 and H2 patients is not significant. The time to recurrence and recurrence patterns were not significant in comparison between each group (Table 2). H1 had less poorly differentiated adenocarcinoma than H2 (p < 0.05) and tended to have a higher rate of adjuvant chemotherapy than H0 (p = 0.08). (Additional file 1: Table S5).

**Table 2:** The pattern of recurrence due to liver metastasis in resected stage III/IV gallbladder cancer patients without peritoneal dissemination or pre-operative jaundice

	Liver metastasis		
	H0	H1	H2
Number of patients	103	9	7
Recurrence	62 (60%)	4 (44%)	5/6 (83%)
Time to recurrence in recurrent cases (months, median)	14.0	6.0	7.0
Liver	23/101 (23%)	1 (11%)	3/6 (50%)
Lymph node	18/101 (18%)	1 (11%)	2/6 (33%)
Local	12/101 (12%)	1 (11%)	0
Dissemination	16/101 (16%)	1 (11%)	1 (17%)
Others	8/101 (8%)	0	0

All were not significant in comparison to each group. H0, no liver metastasis; H1, one liver metastasis; H2, Two or more liver metastasis

### *Univariate analyses of overall survival in patients with resected GBC and liver metastasis (Table **3**)*

**Table 3:** Univariate analyses of overall survival in patients with resected GBC and liver metastasis

		n	3 years OS	p value^a^
Period	2000–2006′/2007–2013′	15/11	7.86/45.5	**0.04**
Age (year)	< 70/≥ 70	15/1	26.7/22.2	0.90
Sex	Male/female	13/13	30.8/18.2	0.90
Preop. jaundice	No/Yes	17/9	40.2/0	0.05
NAC	No/Yes	23/3	28.8/0	0.70
CA19-9, U/L	≤ 37/> 37	8/18	28.6/23.6	1.0
CEA, ng/mL	> 5/≤ 5	15/11	26.7/22.2	0.80
hepatectomy	minor/major	13/13	50.0/0	**< 0.001**
BDR	With/without/PD	15/8/3	14.3/43.8/33.3	0.2
Blood loss, mL	< 864/≥ 864	9/17	71.4/5.88	**< 0.001**
Surgery time, min	< 366/≥ 366/	13/13	45.8/7.69	**0.007**
Histology	Pap/other than pap	5/21	60.0/15.4	**0.05**
AJCC T 8th	T2/ T3,4	8/18	71.4/5.88	**0.002**
AJCC N 8th	N0/N1/N2	9/12/3	22.2/30.7/0/33.3	0.90
P	without /with	23/3	28.6/0	0.03
Residual cancer	R0/R1	16/10	28.7/20.0	0.30
Morbidity*	≤ 2/≥ 3	21/5	31.8/0	**0.09**
AC	with/without	15/10	40.0/0	**0.09**

Bold values indicate *p* < 0.05

OS, overall survival; NAC, Neoadjuvant chemotherapy; CA, cancer antigen; CEA, carcinoembryonic antigen; AJCC, American Joint Committee on Cancer

*Clavien–Dindo classification; AC, Adjuvant chemotherapy. pap/tub1/tub2/tub3, T2/T3/T4, N0/N1/N2, R0/R1,2 (Additional file 1: Table S1)

^a^Log-rank test

According to the univariate analysis of resected patients with GBC with liver metastases (n = 26), the surgery period 2007–2013, those who underwent minor hepatectomy; had less blood loss, less surgery time, papillary adenocarcinoma (vs. other types of adenocarcinoma), and T2 (vs. T3 and 4) exhibited significantly prolonged survival. Morbidity of Clavien–Dindo classification ≤ 2 (vs. ≥ 3) and received adjuvant chemotherapy were marginally not significant.

### Histopathological results: size, number, and distribution of liver metastases in the resected specimens

The median maximum size of resected liver metastases was 5 (range 1.9–35) mm, and the number of liver metastases was 1 in 13 (50%) of the 26 cases, 2 in 6 (23%) cases, 3 in 3 (12%) cases and more than 4 in 4 (15%) cases. The metastatic site was multiple metastases of the right liver in 4 cases and unknown in 3 cases. Of the remaining 19 cases, 16 were segment 4/5, 2 were segment 6, 2 were segment 8, and 1 was segment 3 (There are duplications).

### Long-term survival in patients with GBC and liver metastases

Liver metastases found during surgery or pathological examination were relatively common, and 4 out of 5 patients had T2 disease (Additional file 1: Table S6). These metastases were resected via minor hepatectomy without major postoperative complications, and patients received postoperative adjuvant chemotherapy.

## Discussion

This study examined whether there is a condition that can be called oligometastasis in patients with gallbladder cancer or not, whether surgical treatment is meaningful in these oligometastatic states, and if it is significant, what kind of surgical procedure is desirable. This study indicated that some patients with small single liver metastases might be in an oligometastatic state, and minor hepatectomy and adjuvant chemotherapy may be acceptable.

Hellman et al. [16] proposed the concept of oligometastasis as a state in which some metastases exist before malignant cells acquire widespread metastatic potential [16]. The concept of oligometastasis suggests that if the number of metastases and organ sites is limited, radiation therapy or surgery may cure the condition. Oligometastasis has been reported in various carcinomas [7–10] but not in biliary cancer.

In general, surgery is the only curative treatment for biliary tract cancer that achieves long-term survival. The 5-year overall survival rate for GBC according to the AJCC 8 version is 62.5–78.4% for stage I, 50.2–68.7% for stage II (IIA 68.7%, IIB 81.6%), 25.7–39.7% for stage IIIA, 20.0–22.1% for stage IIIB, 15.7% for stage IVA, and 6.7–12.5% for stage IVB [17, 18]. This study showed better surgical outcomes at all stages compared to the reported data.

In addition, the prognostic factors for resected advanced GBC have been reported to include excessive blood loss [19], poor histology [19], N stage [20], ≥ 4 regional lymph node metastases [19], liver invasion [20], and R1 resection [20]. However, the multivariate analyses of these reports did not include prognostic factors for distant metastasis. In recent years, surgery has been contraindicated in patients with GBC and distant metastases; therefore, the multivariate analyses in these reports [20] rarely included cases of distant metastases.

Patients with biliary cancer who develop distant metastases are considered to have a widespread disease and incurable. Most studies report a 5-year survival rate of 0–6.7%, and long-term survival is rare [21, 22]. Large-scale studies in patients with GBC and distant metastases treated with chemotherapy have reported a median survival of around 7 months [3]. Few patients with GBC and distant metastases achieve 5-year survival after chemotherapy [3, 23]. The median overall survival of patients with locally advanced or metastatic biliary tract cancer receiving chemotherapy with cisplatin and gemcitabine is 11.2–11.7 months [24, 25]. Wakai et al. [26] classified hepatic spread from GBC into three categories: direct invasion through the gallbladder bed, portal tract invasion, and hepatic metastatic nodules. They also reported that direct invasion through the gallbladder bed and portal tract invasion were the main forms of progression of hepatic spread from GBC, and patients with hepatic metastatic nodules had poor outcomes after resection.

However, if pre-operative diagnosis determines that there is no distant metastasis, small liver metastasis is evident around the gallbladder during surgery. In addition, when R0 resection without postoperative morbidity is highly possible using less invasive resection, it is unclear whether it is better to perform resection. Shimizu et al. [27] examined the outcomes of the aggressive surgical management of stage IV GBC. In their study, limited hepatic metastases were resected with the primary tumor only if complete tumor resection was possible. The 5-year survival rate in patients with liver metastasis (n = 16) was 14.4%. However, they mentioned the necessity of clarifying the indications to be considered when performing radical surgery on patients with liver metastasis because of the very limited sample size of their study. In a study of 1526 patients with metastatic gallbladder adenocarcinoma, Yang et al. [5] reported that, while 51.6% of patients had isolated liver metastases, only 5.2% had isolated distant lymph node metastases. Their multivariate analysis showed that not performing surgery at the primary site and not receiving chemotherapy were associated with poor overall survival for patients with isolated liver (HR 1.8 and 2.7, respectively) or distant lymph node metastases (HR 3.6 and 3.7, respectively). Patients who underwent surgery at the primary site showed significantly better overall survival compared to patients not undergoing surgery in both the groups with liver (7 vs. 2 months, p = 0.01) or distant lymph node (12 vs. 6 months, p = 0.01) metastases. The results of the multivariate analysis in this study are consistent with our results.

In recent years, conversion surgery has been gradually reported in biliary cancer. Kato et al. [28, 29] reported that, in patients with initially unresectable locally advanced biliary cancer who received chemotherapy with either gemcitabine (n = 22) or gemcitabine and cisplatin (n = 36), 36.4% and 25.6% of patients, respectively, unresectable has changed to resectable over time and two patients survived for 5 years [28, 29]. Noji et al. [30] described the disease course of 24 patients with biliary cancer, including 12 patients with distant metastases, who were initially ineligible for resection but underwent resection after chemotherapy. Following initial therapy, the 5-year overall survival in these patients was 43.2%. These studies suggest that advances in chemotherapy have led to improved outcomes for unresectable biliary tract cancer.

Liver invasion of 5 mm or more, invasion of the left margin or the entire area of the hepatoduodenal ligament, and four or more regional lymph node metastases are poor prognostic factors for GBC, even in patients without distant metastasis [19, 31].

In our multivariate analysis of stage III/IV GBC, the surgery period 2007–2013 (vs. 2000–2006), pre-operative jaundice, two or more liver metastases (vs. no liver metastasis), and metastasis to the peritoneum (vs. no peritoneal metastasis) were independent prognostic factors for overall survival. Comparing the backgrounds, in surgery period 2007–2013 (vs. 2000–2006), there was less jaundice (25% vs 40%, p = 0.031), lower CEA (2.4 vs 3.8 ng/mL, p = 0.006), more gallbladder bed resection (5% vs. 17%, p = 0.008 [less ≥ 3 segment hepatectomy 31% vs. 41%, p = 0.17), less bleeding (740 vs 1016 mL, p < 0.001), less AJCC T4 (15% vs 28%, p = 0.035), less peritoneal metastasis (4% vs 12%, p = 0.065), and less residual cancer (22% vs 40%, p = 0.008). In other words, the reason why surgery period 2007–2013 was significant is thought to be that the number of cases that progressed between 2007–2013 decreased.

In our univariate analyses of patients with resected GBC and liver metastasis, those who underwent minor hepatectomy, those who had less blood loss, those with less surgery time, those with papillary type histology, those with T2 stage, those with postoperative morbidity of Clavien–Dindo classification ≤ 2, and those who received adjuvant chemotherapy showed more favorable outcomes (Table 3). Four of five patients with liver metastases who survived for 5 years did not have the abovementioned poor prognostic factors at T2 (Additional file 1: Table S6). In addition, four cases were found to have liver metastases during the operation, one case was found to have liver metastases due to postoperative pathology, four cases had a single metastasis (8 mm or less), and one case had two metastases. Therefore, patients with T2 GBC with small liver metastases, first diagnosed during surgery and free of other poor prognostic factors, may be in the oligometastatic state of GBC. Furthermore, in these patients, long-term survival may be expected with minor hepatectomy and R0 with postoperative adjuvant chemotherapy. The next important step will be to conduct a study with a higher number of GBC resections with small liver metastasis resulting in R0 resection (in a minor hepatectomy) with chemotherapy and enroll more long-term survivors.

This study has several limitations. It is a retrospective study, and there were very few H1/H2 cases and multidisciplinary clinical approaches, including pre-operative or postoperative indications. The number of liver metastases was determined intra-operatively through macroscopic findings and ultrasonography; however, it is difficult to accurately determine the number of micro-hepatic metastases using these methods. Furthermore, the surgical procedure and surgical indication to be selected may differ depending on the institution and the surgeon. There may also be different choices of pre-operative and adjuvant chemotherapy.

## Conclusions

For GBC patients with small liver metastases that are initially diagnosed during or after surgery and in the absence of other poor prognostic factors, minor hepatectomy with R0 resection and postoperative chemotherapy may be considered as an option.

## Supplementary Information

**Additional file 1. Table S1.** American Joint Committee on Cancer 8th classification of the Gallbladder with modified definition of the distant metastasis, resectability and histological typing. **Table S2.** Patient characteristics by institutions. **Table S3.** Reasons for non-resected cases by institutions. **Table S4.** The 5-year survival rate for each stage of each facility and the recurrence rate for each facility. **Table S5.** Characteristics of the patients with stage 3 or 4 GBC among H0-2. **Table S6.** Long-term survivors among gallbladder cancer patients with liver metastasis.

## Data Availability

The datasets generated and/or analyzed during the current study are not publicly available due to restrictions on the availability of these data used under the license of the current study but are available from the corresponding author on reasonable request.

## Abbreviations

ACRoS1406: Association for Clinical Research on Surgery Group
AJCC 8: American Joint Committee on Cancer
CA 19.9: Carbohydrate antigen 19.9
CEA: Carcinoembryonic antigen
CT: Computed tomography
H0: No liver metastasis
H1: One liver metastasis
H2: Two or more liver metastases
HR: Hazard ratio
GBC: Gallbladder cancer
No at risk: Number at risk
M1: With distant metastasis
R0: Complete resection with negative margin
R1/2 resection: Grossly negative but microscopically/grossly and microscopically positive margin of resection
T2: Tumor invades the peri-muscular connective tissue on the peritoneal side without the involvement of the serosa or on the hepatic side with no extension into the liver
T3: Tumor perforates the serosa and/or directly invades the liver and/or one other adjacent organ or structure, such as stomach, duodenum, colon, pancreas, omentum, or extrahepatic bile ducts
T4: Tumor invades the main portal vein or hepatic artery or invades two or more extrahepatic organs or structures
Tub1.2: Well or moderately differentiated adenocarcinoma
