# Supplementary material for: Examination of the characteristics of long-term survivors among patients with gallbladder cancer with liver metastasis who underwent surgical treatment: a retrospective multicenter study (ACRoS1406)
Source: BMC Gastroenterol. 2022 Mar 28;22:152. doi: 10.1186/s12876-022-02234-9 (PMC8962041; doi:10.1186/s12876-022-02234-9)
Supplement: Supplementary file 1 — Additional file 1. Table S1. American Joint Committee on Cancer 8th classification of the Gallbladder with modified definition of the distant metastasis, resectability and histological typing. Table S2. Patient characteristics by institutions. Table S3. Reasons for non-resected cases by institutions. Table S4. The 5-year survival rate for each stage of each facility and the recurrence rate for each facility. Table S5. Characteristics of the patients with stage 3 or 4 GBC among H0-2. Table S6. Long-term survivors among gallbladder cancer patients with liver metastasis. [file 12876_2022_2234_MOESM1_ESM.docx]

Supplemental table 1. American Joint Committee on Cancer 8^th^ classification of the Gallbladder with modified definition of the distant metastasis, resectability and histological typing

| T category | |
| --- | --- |
| T2 | Tumor invades the peri-muscular connective tissue on the peritoneal side without involvement of the serosa or on the hepatic side with no extension into the liver |
| T3 | Tumor perforates the serosa and/or directly invades the liver and/or one other adjacent organ or structure, such as stomach, duodenum, colon, pancreas, omentum, or extrahepatic bile ducts |
| T4 | Tumor invades the main portal vein or hepatic artery or invades two or more extrahepatic organs or structures |
| N category | |
| N0 | No metastasis of regional lymph nodes which include along the common bile duct, hepatic artery, portal vein, and cystic duct |
| N1 | One to three positive regional lymph nodes |
| N2 | Four or more positive regional lymph nodes |
| Modified definition of the distant metastasis in this article | |
| Distant LNM | Distant lymph node metastasis |
| H0 or P0 | No liver metastasis or peritoneal metastasis |
| H1 (H2) | One (Two or more) liver metastasis |
| P1 (P2) | Metastasis to peritoneum adjacent to extrahepatic bile duct (A few or numerous metastases to the distant peritoneum) |
| Resectability | |
| R0 | Complete resection with grossly and microscopically negative margin |
| R1 | Grossly negative but microscopically positive margin of resection; |
| R2 | Grossly and microscopically positive margin of resection |
| Histological typing | |
| Pap, papillary adenocarcinoma; tub1, well differentiated adenocarcinoma; tub2, moderately differentiated adenocarcinoma; tub3, poorly differentiated adenocarcinoma | |

Supplemental Table 2. Patient characteristics by institutions

| ^#^Factor | | | Total | KU | SMU | TMDU | YCU | TWMU |
| --- | --- | --- | --- | --- | --- | --- | --- | --- |
|  | | | n = 462 | n = 68 (15%) | n = 25 (5%) | n = 89 (18%) | n = 87 (18%) | n = 193 (43%) |
| Age (y, median) | | | 71 | 70 | 72 | 72 | 71 | 70 |
| Women | | | 237 (51%) | 31 (46%) | 13 (52%) | 46 (52%) | 40 (46%) | 107 (55%) |
| Jaundice | | | 137 (30%) | 11 (16%) | 2 (8%) | 22/85 (26%) | 33 (38%) | 69 (36%) |
| CA19.9 (median, U/ml) | | | 46 (n = 446) | 14 (n = 62) | 22 (n = 24) | 107 (n = 87) | 48 | 54 (n = 186) |
| CEA (median, ng/dl) | | | 3.0 (n = 488) | 2.5 (n = 61) | 1.8 (n = 24) | 3.2 (n = 88) | 3.2 | 3.4 (n = 188) |
|  | | |  |  |  |  |  |  |
| Resected | | | 328 (71%) | 67 (99%) | 20 (80%) | 49 (55%) | 41 (47%) | 151 (78%) |
| Period 2007–2013 | | | 172 (52%) | 33 (49%) | 16 (80%) | 28 (57%) | 22 (54%) | 73 (48%) |
| Preoperative chemotherapy | | | 11 (3.4%) | 1 (1.5%) | 0 | 3 (6.1%) | 5 (12%) | 2 (1.3%) |
| Incidental | | | 54 (16%) | 17 (26%) | 4 (20%) | 8 (16%) | 6 (15%) | 18 (12%) |
| Procedures | | Ch | 110 (34%) | 19 (28%) | 6 (30%) | 21 (43%) | 9 (22%) | 55 (36%) |
|  |  | GB* | 98 (30%) | 31 (46%) | 3 (15%) | 8 (16%) | 19 (46%) | 37 (24%) |
|  |  | S4aS5 | 50 (15%) | 6 (9%) | 8 (40%) | 9 (18%) | 7 (17%) | 20 (13%) |
|  |  | hepatectomy (≥3 seg) | 62 (21%) | 11 (16%) | 3 (15%) | 11 (22%) | 6 (15%) | 39 (26%) |
| PD or BDR | | without | 171 (52%) | 48 (72%) | 8 (40%) | 24 (50%) | 20 (49%) | 71 (47%) |
|  | | BDR | 125 (38%) | 12 (18%) | 11 (55%) | 19 (40%) | 19 (46%) | 64 (42%) |
|  | | PD | 31 (10%) | 7 (10%) | 1 (5%) | 5 (10%) | 2 (5%) | 16 (11%) |
| Vascular resection | | | 25 (8%) | 4 (6%) | 0 | 0 | 4 (10%) | 17 (11%) |
| *AJCC^8th^ Tis or 1/T2/T3/T4 | | | 72/112/102/41 | 21/27/13/6 | 3/3/14/0 | 10/19/11/8 | 9/16/7/9 | 29/47/57/18 |
| *AJCC^8th^ N0/N1/N2/distant | | | 200/76/14/37 | 51/10/1/5 | 16/3/0/1 | 27/13/3/5 | 26/9/1/5 | 80/41/9/21 |
| AJCC^8th^ M1 | | | 54 (17%) | 6 (9%) | 2 (10%) | 6 (13%) | 4 (10%) | 36 (24%) |
| *R0 | | | 262/325 (81%) | 57 (85%) | 17 (85%) | 35/47 (75%) | 38/40 (95%) | 115 (76%) |
| Morbidity (CD ≥3) | | | 64/330 (19%) | 11/67 (16%) | 4 (20%) | 6/50 (12%) | 9 (22%) | 41 (22%) |
| Mortality | | | 5 (1.5%) | 0 | 0 | 1(2%) | 1 (2.4%) | 3 (2%) |
| Adjuvant chemotherapy | | | 94/319 (30%) | 18/66 (27%) | 5/19 (26%) | 13/45 (29%) | 16 (39%) | 42/148 (28%) |

*see Supplemental Table 1. KU, Keio university; SMU, St Marianna university; TMDU, Tokyo Medical Dental University; YCU, Yokohama City University; TWMU, Tokyo Women’s Medical University; CEA, carcinoembryonic antigen; CA, carbohydrate antigen; Ch, cholecystectomy; GB, gallbladder bed resection; S4aS5, resection of segment 4a and 5 of the liver; BDR, bile duct resection; PD, pancreatoduodenectomy; AJCC, [American Joint Committee on Cancer](https://cancerstaging.org/); R0, complete resection with negative margin; CD, Clavien-Dindo

Supplemental Table 3. Reasons for non-resected cases by institutions

|  | n = 134 | n = 1 | n = 5 | n = 40 | n = 46 | n = 42 |
| --- | --- | --- | --- | --- | --- | --- |
| Liver met | 41/132 (31%) | 0 | 0 | 15 (38%) | 9 (20%) | 17 (40%) |
| Peritoneum met | 22/132 (17%) | 0 ( | 0 | 10 (25%) | 6 (13%) | 6 (14%) |
| Local met | 63/132 (48%) | 1 (100%) | 4 (80%) | 21 (53%) | 19 (41%) | 18 (43%) |
| Lung met | 5/132 (3.7%) | 0 | 0 | 1 (2.5%) | 3 (6.5%) | 1 (2,4%) |
| Distant lymph node met | 36/132 (27%) | 0 | 1 (20%) | 16 (40%) | 9 (20%) | 10 (24%) |
| General condition | 6/132 (5%) | 0 | 0 | 1 (2.5%) | 1 (2.2%) | 4 (10%) |

KU, Keio university; SMU, St Marianna university; TMDU, Tokyo Medical Dental University; YCU, Yokohama City University; TWMU, Tokyo Women’s Medical University; met, metastasis

Supplemental Table 4. The 5-year survival rate for each stage of each facility and the recurrence rate for each facility

|  | | Total | KU | SMU | TMDU | YCU | TWMU |
| --- | --- | --- | --- | --- | --- | --- | --- |
| Number | | n = 462 | n = 68 (15%) | n = 25 (5%) | n = 89 (18%) | n = 87 (18%) | n = 193 (43%) |
| ^#^Overall 5y survival (%), resected | |  |  |  |  |  |  |
| AJCC 8th | Stage I | 91% (n=72) | 89% (n=21) | 67% (n=3) | 100% (n=10) | 88% (n=9) | 95% (n=29) |
|  | Stage II | 80% (n=63) | 87% (n=23) | 100% (n=2) | 67% (n=9) | 78% (n=9) | 79% (n=20) |
|  | Stage IIIa | 51% (n=37) | 33% (n=5) | ^♦†^81% (n=11) | ^♦^0 (n=4) | ^†‡^0 (n=3) | ^‡^54% (n=14) |
|  | Stage IIIb | 33% (n=46) | 40% (n=5) | 50% (n=2) | 22% (n=9) | 30% (n=5) | 37% (n=25) |
|  | Stage IVa | 24% (n=27) | 50% (n=4) | NA (n=0) | 25% (n=4) | 17% (n=6) | 23% (n=13) |
|  | Stage IVb | 20% (n=83) | ^♦^0 (n=9) | ^♦^0 (n=2) | ^†^0 (n=13) | 22% (n=9) | ^†^26% (n=50) |
| ^$^Recurrence rate | | 145/327 (44%) | ^♦†^19 (28%) | 7 (35%) | 22 (45%) | ^♦^22 (54%) | ^†^75/150 (50%) |

^#^Log-rank tests, ^$^2x5 fisher exact test. ^♦†‡^p<0.05 between two groups. KU, Keio university; SMU, St Marianna university; TMDU, Tokyo Medical Dental University; YCU, Yokohama City University; TWMU, Tokyo Women’s Medical University; CEA, carcinoembryonic antigen; CA, carbohydrate antigen; Ch, cholecystectomy; GB, gallbladder bed resection; S4aS5, resection of segment 4a and 5 of the liver; BDR, bile duct resection; PD, pancreatoduodenectomy; AJCC, [American Joint Committee on Cancer](https://cancerstaging.org/); R0, complete resection with negative margin; CD, Clavien-Dindo

Supplemental Table 5. Characteristics of the patients with stage 3 or 4 GBC among H0-2

|  | With or without liver metastasis | | |  |
| --- | --- | --- | --- | --- |
|  | H0 | H1 | H2 |  |
| Number | n = 103 | n = 9 | n = 7 |  |
| ^♦^Period (2007–2013) | 59 (57%) | 5 (56%) | 4 (57%) |  |
| ^#^Age (y, median) | 72.0 | 69.0 | 72.0 |  |
| ^♦^Women | 59 (57%) | 4 (44%) | 4 (57%) |  |
| ^♦^Preoperative jaundice | 55 (28%) | 4 (25%) | 6 (32%) |  |
| ^#^CA19-9 (U/L, median) | 28 | 61 | 16 |  |
| ^#^CEA (ng/mL, median) | 2.4 | 3.4 | 7.2 |  |
| ^Ψ^Hepatectomy (GB/Ch/S4aS5/≥3 segments) | 36/24/28/15 | 3/0/3/3 | 2/0/3/2 |  |
| ^∀^PD/with BDR/without BDR | 12/48/43 | 1/4/4 | 0/4/3 |  |
| ^♦^Vascular resection | 5 (4.9%) | 1 (11%) | 1 (14%) |  |
| ^#^Blood loss (g, median) | 700 | 560 | 700 |  |
| ^#^Surgery time (min, median) | 342 | 301 | 278 |  |
| ^∀^Histology pap/tub1.2/tub3 | 13/66/24 | *2/7/0 | *1/2/4 |  |
| ^∀^AJCC T2/T3/T4 | 36/53/14 | 5/4/0 | 3/3/1 |  |
| ^Ψ^AJCC N0/N1/N2/distant LNM | 33/43/8/19 | 3/5/0/1 | 2/4/0/1 |  |
| ^♦^R0 | 80/101 (79%) | 7 (78%) | 5 (71%) |  |
| ^♦^Morbidity Clavien–Dindo classification ≥3 | 27/102 (26%) | 0 | 2 (29%) |  |
| ^♦^Adjuvant chemotherapy | ^φ^44/97 (45%) | ^φ^7 (78%) | 3 (43%) |  |

*p<0.05 between two group. ^#^Wilcoxon rank sum test. ^♦^2x2, ^∀^3x2, ^Ψ^4x2, Fisher exact test. ^φ^P-value of comparison about adjuvant chemotherapy between H0 and H1 was 0.08. No mark indicates no significance between the other 2 groups. CA, carbohydrate antigen; CEA, carcinoembryonic antigen; Ch, cholecystectomy; GB, gallbladder bed resection; S4aS5, resection of segment 4a and 5 of the liver; PD, pancreatoduodenectomy; BDR, bile duct resection; AJCC, [American Joint Committee on Cancer](https://cancerstaging.org/). pap/tub1/tub2/tub3, T2/T3/T4, N0/N1/N2/Distant LNM, H0/H1/H2, P0/P1/P2, R0 (See Supplemental table 1).

Supplemental Table 6. Long-term survivors among gallbladder cancer patients with liver metastasis

| Age/  Sex | Metastatic  site | Timing of  diagnosis  of LM | Size (mm) | | Surgical  Procedure | Surgical morbidity  (CD ≥ 3) | AJCC 8^th^ | | | R | Neoadjuvant/Adjuvant chemo | Outcome | Survival time  (months) |
| --- | --- | --- | --- | --- | --- | --- | --- | --- | --- | --- | --- | --- | --- |
|  |  |  | Metastasis  (number) | Primary site |  |  | T | N | M |  |  |  |  |
| 60sM | Liver, S5 | DS | 8 (1) | 130 | GB | No | 2 | 1 | 1 | 0 | No/S1 | Alive with rec | 60 |
| 70sM | Liver, S4 | DS | 3 (1) | 30 | S45, BDR | No | 2 | 1 | 1 | 0 | No/UFT | Alive without rec | 144 |
| 70sM | Liver, S4 | DS | 3 (1) | 35 | GB | No | 2 | 0 | 1 | 0 | No/Gem→S1 | Alive without rec | 69 |
| 60sF | Liver, S4, | PE | 5 (1) | 30 | GB, PD | No | 3 | 2 | 1 | 1 | No/Gem | Alive without rec | 126 |
| 50sM | Liver, S5 | DS | 12 (2) | 55 | S45 | No | 2 | 0 | 1 | 1 | No/S1 | Alive without rec | 115 |

LM, liver metastasis; CD, Clavien-Dindo; AJCC, American Joint Committee on Cancer; R, resectability; S, segment; DS, during surgery; PE, pathological examination; GB, gallblader bed resection; S45, resection of segment 4a and 5 of the liver; PD, pancreatoduodenectomy; BDR, bile duct resection; S1, Tegafur, Gimeracil, Oteracil Potassium; UFT, Tegafur, Uracil; Gem, Gemcitabin
